# Standardised Transparent Orthopaedic Reporting and Modelling for AI (STORM‐AI)—Guidelines for reporting artificial intelligence studies in orthopaedics from the ESSKA AI Working Group

**DOI:** 10.1002/jeo2.70702

**Published:** 2026-03-30

**Authors:** Felix C. Oettl, Bálint Zsidai, Yinan Yu, James Pruneski, Thomas Tischer, Ayoosh Pareek, Alberto Grassi, Stefano Zaffagnini, Michael T. Hirschmann, Kristian Samuelsson, ESSKA Artificial Intelligence Working Group

**Affiliations:** ^1^ Department of Orthopedic Surgery Balgrist University Hospital, University of Zürich Zurich Switzerland; ^2^ Department of Orthopaedics, Institute of Clinical Sciences, Sahlgrenska Academy University of Gothenburg Gothenburg Sweden; ^3^ Sahlgrenska Sports Medicine Center Göteborg Sweden; ^4^ Department of Computer Science and Engineering Chalmers University of Technology Gothenburg Sweden; ^5^ Department of Orthopaedic Surgery Tripler Army Medical Center Honolulu Hawaii USA; ^6^ Department of Orthopaedic Surgery Universitymedicine Rostock Rostock Germany; ^7^ Department of Orthopaedic and Trauma Surgery Malteser Waldkrankenhaus Erlangen Erlangen Germany; ^8^ Hospital for Special Surgery New York New York USA; ^9^ Clinica Ortopedica e Traumatologica IRCCS Istituto Ortopedico Rizzoli Bologna Italy; ^10^ Department of Orthopaedic Surgery and Traumatology Kantonsspital Baselland Bruderholz Switzerland; ^11^ University of Basel Basel Switzerland; ^12^ Department of Health and Rehabilitation, Institute of Neuroscience and Physiology, Sahlgrenska Academy University of Gothenburg Gothenburg Sweden

**Keywords:** artificial intelligence, guidelines, machine learning, orthopaedics, reporting standards

## Abstract

**Purpose:**

The rapid growth of Artificial Intelligence (AI) in orthopaedic research has led to inconsistencies in study reporting, hindering evaluation and clinical translation. This initiative aimed to develop the STORM‐AI (Standardised Transparent Orthopaedic Reporting and Modelling‐AI) guidelines to enhance the transparency, completeness, and quality of reporting for AI studies in orthopaedics.

**Methods:**

The ESSKA AI Working Group, a multinational and multidisciplinary team of experts, developed the STORM‐AI guidelines through a multi‐step consensus process. This involved a comprehensive review of existing AI reporting standards (e.g., CONSORT‐AI, STARD‐AI and TRIPOD), followed by iterative rounds of drafting, review, and refinement to incorporate orthopaedic‐specific considerations.

**Results:**

The consensus process resulted in the STORM‐AI checklist and an accompanying Explanation and Elaboration (E&E) document. The guidelines provide specific reporting recommendations across all study sections, including study design, data characteristics, model development, performance metrics, ethical considerations and clinical workflow integration. Key areas of emphasis include rigorous validation, clear outcome definition, and error analysis within the orthopaedic context.

**Conclusion:**

The STORM‐AI guidelines provide a crucial framework for authors, reviewers, and journals to improve the evidence base for AI in orthopaedic care. Widespread adoption is anticipated to foster more robust, reproducible, and clinically valuable innovations, facilitating the responsible integration of AI into orthopaedics.

**Level of Evidence:**

Level V.

**Abbreviations**
AI: artificial intelligence
CI: confidence interval
CNN: Convolutional Neural Network
CONSORT: Consolidated Standards of Reporting Trials‐Artificial Intelligence
DSC: dice similarity coefficient
EHR: Electronic Health Records
EQUATOR: Enhancing the QUAlity and Transparency Of health Research
ESSKA: European Society of Sports Traumatology, Knee Surgery and Arthroscopy
E&E: explanation and elaboration
LIME: local interpretable model‐agnostic explanations
MCC: Matthews Correlation Coefficient
MCID: minimal clinically important differences
NICE: National Institute for Health and Care Excellence (NICE)
NLP: natural language processing
NPV: negative predictive value
PACS: picture archiving and communication systems
PPV: positive predictive value
PROMs: patient‐reported outcome measures
ROC: receiver operating characteristic
STARD: Standards for Reporting of Diagnostic Accuracy Studies
STORM: Standardised Transparent Orthopedic Reporting and Modelling
TRIPOD: Transparent Reporting of a multivariable prediction model for Individual Prognosis or Diagnosis
XAI: eXplainable AI

## INTRODUCTION

The integration of artificial intelligence (AI) into orthopaedic surgery and musculoskeletal medicine is rapidly accelerating. Over the past decade, and particularly in recent years, there has been an exponential increase in publications exploring AI applications across the orthopaedic spectrum [11, 12]. These range from diagnostic aids using medical imaging for fracture detection or osteoarthritis grading, predictive modelling for patient‐reported outcome measures (PROMs) and implant survivorship, to intraoperative guidance systems and personalised treatment planning [11].

However, this rapid proliferation of AI research in orthopaedics has also highlighted significant challenges, primarily stemming from a lack of standardised reporting. Many published studies suffer from methodological heterogeneity and incomplete documentation of key aspects related to data curation, model development, and performance evaluation [3, 4]. This often results in research of varying quality, making it difficult for clinicians, researchers, and peer reviewers to evaluate the validity of the findings, compare results across different studies, or reproduce the work [4, 5]. Such deficiencies not only impede scientific progress but also hinder the crucial step of translating promising AI models from research prototypes into reliable and ethically sound clinical tools that can benefit patients [2]. The 'black box' nature of some AI algorithms, where the decision‐making process is opaque even to developers, coupled with inadequate reporting on model validation and generalisability, further complicates clinical acceptance and trust [2, 3, 9].

Without transparent and comprehensive reporting, the orthopaedic community cannot effectively scrutinise the potential biases, limitations, or true clinical utility of proposed AI solutions. Recognising these challenges, several general reporting guidelines for AI research have been developed, such as Consolidated Standards of Reporting Trials‐Artificial Intelligence (CONSORT‐AI) for clinical trials, Standards for Reporting of Diagnostic Accuracy Study (STARD‐AI) for diagnostic accuracy studies, and Transparent Reporting of a multivariable prediction model for Individual Prognosis or Diagnosis (TRIPOD) for multivariable prediction models [4, 6, 13]. While these guidelines provide an invaluable foundation, the unique nature of orthopaedic data (e.g., specific imaging modalities and artifacts, biomechanical parameters, implant‐related considerations, diverse surgical procedures, and distinct patient populations) necessitates a specialised adaptation to ensure all domain‐specific nuances are adequately addressed.

To address these orthopaedic‐specific challenges, the European Society of Sports Traumatology, Knee Surgery and Arthroscopy (ESSKA) established the AI Working Group. This group comprises a multinational and multidisciplinary team of orthopaedic surgeons, data scientists, and medical researchers with expertise in artificial intelligence. The group′s mission is to develop evidence‐based guidance to support the high‐quality and responsible integration of AI into orthopaedic and musculoskeletal medicine.

This group has undertaken the development of guidelines for Standardised Transparent Orthopaedic Reporting and Modelling in artificial intelligence (STORM‐AI). The primary aim of STORM‐AI is to enhance the quality, transparency, and completeness of reporting for AI research in orthopaedic surgery and related fields. By providing a tailored checklist and an accompanying explanation and elaboration (E&E) document, STORM‐AI seeks to:

1. Guide researchers in the robust design, conduct, and comprehensive reporting of their AI studies in orthopaedics.
2. Assist journal editors and peer reviewers in the critical appraisal of submitted manuscripts.
3. Enable clinicians and readers to better understand, interpret, and evaluate the evidence for AI applications.
4. Promote reproducibility and the potential for independent validation of AI research in orthopaedics.
5. Facilitate the responsible development and clinical integration of effective and reliable AI tools to improve patient care in orthopaedics.

These guidelines are intended to complement, not replace, existing broader AI reporting standards by offering orthopaedic‐specific extensions and clarifications, drawing upon the principles of CONSORT‐AI, STARD‐AI and TRIPOD.

## METHODS

The STORM‐AI guidelines were developed through a systematic, multi‐step consensus process by the ESSKA AI Working Group, representing an approach that parallels yet diverges meaningfully from the broader ecosystem of AI reporting guidelines. Like TRIPOD, CONSORT‐AI and other EQUATOR‐endorsed frameworks, the methodology was designed to ensure the final recommendations were evidence‐informed, relevant to the specialty, and grounded in expert agreement. The development process began with a comprehensive review of existing, widely accepted reporting guidelines for AI‐related research as the foundational architecture. This approach mirrors the strategy employed by other emerging specialty‐specific guidelines like NICE guidance (for health technology assessment), which similarly build upon established EQUATOR frameworks rather than developing standards de novo.

What distinguishes STORM‐AI is its recognition that orthopaedic‐specific reporting challenges were inadequately addressed by these general frameworks. In parallel with synthesising core elements from existing guidelines, the working group conducted a targeted literature review of published AI studies in orthopaedic surgery to identify common reporting deficiencies and unique, specialty‐specific requirements not adequately captured by general guidelines. These orthopaedic‐specific challenges are distinct from challenges identified in other specialty adaptations. This represents a more granular domain‐specialisation approach compared to broader frameworks.

The working group then engaged in a multi‐step, iterative consensus process to refine the initial list of items, with an initial draft checklist compiled and circulated to all members. Refinement occurred over several months through serial feedback rounds. During this refinement process, each proposed item was evaluated against three explicit criteria: relevance,clarity, and feasibility, criteria that align with those employed by other guideline development initiatives but emphasise the important dual‐audience consideration of orthopaedic surgery (surgeon–data scientist collaboration). Items from the foundational guidelines were systematically adapted to the orthopaedic context, and items specific to orthopaedics were proposed, debated, and incorporated. The checklist underwent iterative revision until agreement was reached on the inclusion, wording, and placement of every item. Notably, STORM‐AI positions itself as aligned with living document principles, acknowledging the rapidly evolving nature of orthopaedic AI research and anticipating periodic updates as technological innovations and best practices emerge within the specialty.

## RESULTS: EXPLANATION AND ELABORATION

This document provides a detailed explanation and elaboration for each Part in the STORM‐AI checklist. The aim of STORM‐AI is to improve the transparency, completeness, and quality of reporting for studies focusing on the development, validation, or application of AI in orthopaedic surgery and musculoskeletal medicine. By providing this guidance, we intend to assist authors, peer reviewers, and editors in ensuring that published research is understandable, reproducible, and allows for critical appraisal of an AI model′s utility and limitations in the orthopaedic context. Examples for each part of the checklist are provided in the supplementary material.

## I. TITLE AND ABSTRACT

The title and abstract are essential for enabling readers to quickly understand a study′s content and relevance, and for ensuring correct indexing in literature databases.

### Part 1A: Identification as a study of an AI model in orthopaedics

The title should clearly and unambiguously identify the article as research concerning an artificial intelligence model and specify its application within an orthopaedic context. This fundamental piece of information allows for efficient literature searches, correct indexing by databases (e.g., PubMed and Embase), and helps readers, clinicians, and researchers to rapidly determine the relevance of the manuscript to their interests. Clear identification is the first step towards transparent reporting, which is paramount for assessing the reliability and potential impact of AI systems in medicine.

### Part 1B: Specific orthopaedic problem/application addressed

Beyond generally identifying the study as AI in orthopaedics, the title and/or abstract should clearly state the specific clinical orthopaedic question, task, or problem the AI model is designed to address. This provides immediate context about the model′s intended use and its potential clinical or research niche. Specificity here helps in understanding the scope and relevance of the AI application.

### Part 1C: Study objectives

The abstract (and the title, if space permits and clarity is maintained) must explicitly state the main objective(s) of the study. This includes clearly indicating whether the research focuses on the initial development of a new AI model, the validation (internal or external) of a previously developed model, an update or refinement of an existing model, or a direct comparison of different AI models or an AI model against human experts or conventional methods. Stating the study type is crucial for interpreting its findings and contribution to the evidence base.

### Part 1D: Mention of model type

The abstract should briefly specify the nature or functional type of the AI model or the task it performs. Common types in orthopaedics include diagnostic models (e.g., classifying fracture vs. no fracture), prognostic models (e.g., predicting future risk of implant failure or patient outcome), image segmentation models (e.g., delineating bone or cartilage boundaries), or natural language processing models for extracting information from clinical notes. This information helps readers categorise the AI application and understand its technical underpinnings.

## II. INTRODUCTION

The Introduction section should provide the necessary context, articulate the rationale for the study, and clearly state its objectives and intended contribution.

### Part 2A: Scientific and clinical background, including intended use of the AI

This part of the introduction should provide a concise yet comprehensive overview of the specific problem being addressed. It should detail the current clinical challenges, describe existing diagnostic or prognostic methods and their limitations, and clearly outline the unmet need or specific opportunity that the AI model is intended to address. Crucially, authors should describe the intended use of the AI system within a realistic clinical or research workflow. This helps readers understand the clinical relevance and potential impact of the study. The lack of clear reporting on intended use can be a barrier to translation.

### Part 2B: Specific objectives and hypotheses, clearly stating if it′s development, validation or both

Following the background, authors must clearly articulate the primary and any secondary objectives of their study. If applicable, specific, testable hypotheses should be stated. It is essential to reiterate here (even if mentioned in the abstract) whether the study′s primary aim is to develop a new AI model, to validate an existing model (and specify the type of validation, e.g., internal, external, temporal, geographical), to update or extend an existing model, or to compare the performance of different AI models or an AI model against current clinical standards or human experts. Precisely stated objectives are fundamental for assessing whether the study design and results adequately address the research question. This clarity is vital for building a robust evidence base for AI in orthopaedics.

### Part 2C: Intended population and setting

Authors should clearly define the specific patient population (e.g., demographics, specific orthopaedic condition, stage of disease, inclusion of specific implants and prior treatments) and the clinical setting(s) (e.g., emergency department, primary care clinic, specialised academic orthopaedic center, multicenter research consortium and public joint registry) for which the AI model is ultimately intended or was developed/validated. This information is critical for readers to assess the model′s applicability, potential generalisability to their own patients or settings, and to understand the context of the study. AI models are often sensitive to variations in populations and settings, making this explicit reporting essential.

## III. METHODS

The Methods section should provide a clear and detailed description of how the study was designed, conducted, and analysed. For AI studies, this includes comprehensive information about the data used, how the AI model was developed and evaluated, and the statistical methods employed. Transparency in methods is fundamental for reproducibility and critical appraisal.

### Part 3A: Study design

#### Part 3A.i: Prospective or retrospective

Clearly state whether the study was conducted prospectively (data collected specifically for the study moving forward) or retrospectively (using existing data). This distinction is critical as it has implications for potential biases, data quality, and the type of claims that can be made. Prospective studies, especially for validation, are generally considered to provide stronger evidence as they allow for predefined data collection protocols and can better mimic real‐world application. Retrospective studies are common for initial model development and validation but may be subject to selection bias or biases related to data documentation practices at the time of collection.

#### Part 3A.ii: Description of the study population and period

Provide a concise description of the overall population from which the study participants or data were drawn and specify the dates or time period of data collection or participant recruitment. This helps to contextualise the study sample and allows readers to assess the relevance of the findings to other time periods or populations.

#### Part 3A.iii: Setting

Describe the setting(s) where the data were collected or participants were recruited. This could include the type of institution (e.g., academic medical center, community hospital and private clinic), the number of centres involved (single vs. multicenter), and the geographical location(s) if relevant. The setting can significantly influence patient characteristics, data quality, imaging equipment, and treatment protocols, all of which can affect AI model performance and generalisability. Multicenter studies often provide more robust evidence of generalisability.

### Part 3B: Participants/data source

#### Part 3B.i: Eligibility criteria (inclusion/exclusion) for patients/data

Precisely define the criteria used to select participants (for prospective studies) or data instances (for retrospective studies). Inclusion criteria define the target population, and exclusion criteria identify individuals or data points removed to reduce heterogeneity, avoid confounders, or ensure data quality. Clearly reported eligibility criteria are essential for understanding the characteristics of the study sample, assessing the applicability of the model to other populations, and enabling other researchers to potentially replicate the study or compare findings.

#### Part 3B.ii: Source of data

Specify the origin and type of data used to develop or validate the AI model. This could include Picture Archiving and Communication Systems (PACS) for images, Electronic Health Records (EHRs) for clinical data, specialised orthopaedic registries, research databases, or data from wearable sensors or motion capture systems. The source of data influences its structure, quality, and potential biases.

#### Part 3B.iii: Methods of data collection and any pre‐processing specific to orthopaedic data

Describe how the data were collected and any initial pre‐processing steps undertaken before the data were fed into the AI model. It may include details on image acquisition protocols, image normalisation or standardisation techniques (e.g., DICOM windowing/leveling, intensity scaling, image resizing), methods to handle or mitigate imaging artifacts from metallic implants, specific annotation protocols for images (e.g., by orthopaedic surgeons or radiologists), or methods for extracting structured data from unstructured text like operative notes using Natural Language Processing (NLP) if these are inputs to a subsequent model. These details are critical for reproducibility and understanding potential sources of variability or bias.

### Part 3C: Outcome definition

This section is particularly relevant for studies developing or validating prognostic or diagnostic prediction models but defining the target for the AI is crucial in most AI applications.

#### Part 3C.i: Clear definition of the outcome(s) being predicted

Authors must provide a precise and unambiguous definition of the outcome(s) that the AI model is intended to predict or identify. This includes specifying what constitutes the outcome, how it was defined (e.g., using established clinical criteria, registry definitions, specific patient‐reported outcome measure thresholds), and any nuances in its interpretation. For prognostic models, the outcome is the event or state being predicted in the future. For diagnostic models, it is the presence or absence of the target condition. Vague outcome definitions can lead to misinterpretation of the model′s performance and applicability.

#### Part 3C.ii: How and when outcomes were assessed, including follow‐up duration

Describe the methods used to ascertain the outcome(s) and the timing of these assessments. This includes detailing the source of outcome information (e.g., patient records, registries, direct patient contact and specific imaging modalities), who assessed the outcome (e.g., orthopaedic surgeons, radiologists and research coordinators), and whether assessors were blinded to predictor information or AI model output. For prognostic models, the length of follow‐up is critical and must be clearly stated and justified, as the risk of many orthopaedic outcomes changes over time.

#### Part 3C.iii: Any cut‐offs used for continuous outcomes

If a continuous outcome measure (e.g., a PROM score, a radiographic measurement) was dichotomised or categorised for the purpose of model development or evaluation (e.g., to define “improvement” vs. “no improvement,” or “mild” vs. “moderate” vs. “severe”), the specific cut‐off value(s) used must be clearly stated and justified. The rationale for choosing these cut‐offs (e.g., based on established minimal clinically important differences (MCID), receiver operating characteristic (ROC) curve analysis, or literature precedents) should be provided. Dichotomising continuous variables can lead to loss of information, so the choice needs careful consideration and transparent reporting.

### Part 3D: Reference standard

This section is crucial for studies evaluating the accuracy of AI models designed for diagnostic or detection tasks (e.g., identifying fractures, classifying tumour types and detecting implant loosening).

#### Part 3D.i: The “ground truth” used to establish the presence/absence or extent of the condition

Clearly and explicitly describe the reference standard used to confirm the true status of the orthopaedic condition that the AI model is designed to detect or classify. The reference standard is the best available method for establishing “ground truth” and serves as the benchmark against which the AI model′s performance is judged. The choice of reference standard directly impacts the interpretation of the AI model′s accuracy.

#### Part 3D.ii: Rationale for choosing the reference standard

Provide a justification for why the chosen reference standard is considered appropriate for the study and the specific condition. This may involve citing literature that establishes its accuracy, its common acceptance in clinical practice, or its feasibility within the study context. If the reference standard itself has known limitations, these should be acknowledged.

#### Part 3D.iii: Blinding of assessors to AI results and other clinical information

Describe whether the individuals interpreting the reference standard were blinded to the output of the AI model and to other clinical information that was not part of the reference standard itself (e.g., other test results, patient symptoms if the reference is purely image‐based). Similarly, if human readers are being compared to the AI, state whether they were blinded to the AI results and the reference standard. Blinding is crucial to prevent review bias, where knowledge of other findings could influence the interpretation of the reference standard or the index test (AI model).

### Part 3E: Predictors/input data

This section focuses on the data elements that are used as input for the AI model.

#### Part 3E.i: Detailed description of all input data used by the AI

Authors must provide a comprehensive list and detailed description of all variables or data types used as input (predictors or features) for the AI model. This includes demographic information (e.g., age and sex), clinical variables (e.g., BMI, comorbidities, symptoms, duration of symptoms and PROMs), specific imaging features (e.g., raw pixel/voxel data from X‐rays/CT/MRI, pre‐defined radiographic measurements and radiomic features), laboratory values, intraoperative data (e.g., surgical time, implant details if used as input and ligament tension), or biomechanical parameters (e.g., gait analysis metrics and joint angles from sensors). The level of detail should be adequate for another researcher to understand precisely what information the model utilised.

#### Part 3E.ii: How and when predictors/input data were measured/extracted

For each input variable or data type, describe how it was measured or extracted, the timing of its collection relative to the clinical course or outcome, and any instruments or protocols used. For example, if PROMs are used, specify the exact PROM instrument and when it was administered. If radiographic angles are used, describe how they were measured (manually, semi‐automatically) and by whom. If data are extracted from EHRs, describe the extraction process. This detail is vital for assessing the reliability and consistency of the input data.

#### Part 3E.iii: Handling of missing data

Missing data are a common issue in clinical research, including orthopaedic AI studies. Authors must report the extent of missing data for each predictor considered or used in the AI model. Crucially, they must describe the methods used to handle any missing data, such as complete case analysis (listwise deletion), single imputation (e.g., mean, median, regression imputation, if imputation is used it is crucial to provide the respective code snippet as this is a part of the workflow frequently leading to data leakage if not performed correctly), multiple imputation, or if the AI algorithm itself can inherently handle missing values. The choice of method can significantly impact model performance and bias, so transparency is essential. If no data were missing, this should also be explicitly stated.

#### Part 3E.iv: Specific radiographic measurements used, details of segmentation (if input to another model), specific PROMs

This emphasises the need for granular detail when describing orthopaedic‐specific predictors. If specific, named radiographic measurements are used (e.g., alpha angle, tibial slope and Kellgren–Lawrence grade), these should be clearly defined, including how they were derived if not standard. If the output of one AI model (e.g., a segmentation of a bone or cartilage) is used as an input feature for a subsequent AI model (e.g., a prognostic model), the details and performance of that initial segmentation model should be briefly summarised or referenced. Similarly, if PROMs are used, the exact instrument (e.g., KOOS, HOOS, SF‐36 and EQ‐5D) and any specific subscales or summary scores should be reported.

### Part 3F: AI model development and training

This is the core technical section describing how the AI model itself was created and trained.

#### Part 3F.i: Description of the AI model architecture

Provide a clear and sufficiently detailed description of the AI model′s architecture. For deep learning models like Convolutional Neural Networks (CNNs), this includes specifying the type of network (e.g., ResNet50, U‐Net and VGG16), number of layers, types of layers (convolutional, pooling, fully connected), activation functions, and any significant modifications to standard architectures. For traditional machine learning models, specify the algorithm used (e.g., logistic regression, support vector machine, random forest and gradient boosting). If a novel architecture is proposed, more extensive details and diagrams may be necessary. The goal is to provide enough information for another researcher to understand the model′s structure and potentially replicate it.

#### Part 3F.ii: Data pre‐processing steps not covered in 3B

Describe any data pre‐processing or transformation steps applied to the input data specifically for the model training process, beyond the initial data collection and cleaning stages covered in Section III.B. For image‐based AI in orthopaedics, this often includes data augmentation techniques (e.g., rotation, scaling, flipping, adding noise, elastic deformations and contrast adjustments) used to increase the diversity of the training set and improve model robustness. Also, report other steps like feature scaling (e.g., normalisation and standardisation) for numerical inputs, or specific text cleaning for NLP.

#### This Part 3F.iii: Data partitioning

is a critical aspect of AI model development and evaluation. Authors must clearly describe how the available data were partitioned into distinct datasets for training, validation (also sometimes called tuning or development set, used for hyperparameter optimisation and model selection), and testing. Report the exact number of samples (e.g., patients, images and cases) in each set. Specify how the split was performed (e.g., random split, stratified split, temporal split and site‐based split). Crucially, indicate whether the test set was an *internal test set* (drawn from the same underlying population/dataset as the training data but held out) or an *external test set* (data from a different time period, different institution(s), different geographical location, or different patient population). External validation provides stronger evidence of generalisability.

#### Part 3F.iv: Details of the training process

Provide sufficient detail about the model training procedure to allow for replication. This includes specifying the loss function minimised during training (e.g., binary cross‐entropy for classification, mean squared error for regression and Dice loss for segmentation), the optimisation algorithm used (e.g., Adam, SGD and RMSprop), key hyperparameters (e.g., learning rate, batch size, number of epochs and regularisation parameters), and the software frameworks or libraries used (e.g., TensorFlow, PyTorch, Scikit‐learn and R packages with version numbers). For complex models, referencing a public code repository can be invaluable.

#### Part 3F.v: Approach to model selection or feature selection

If applicable, describe the methods used for selecting the final model from several candidates and/or for selecting the most relevant predictors/features from a larger set. This might involve techniques like stepwise selection, LASSO regression for feature shrinkage, recursive feature elimination, or choosing the model architecture or hyperparameter set that performed best on the validation set according to a pre‐specified metric (e.g., highest AUC and lowest log‐loss).

#### Part 3F.vi: For clinical trials: Description of the AI intervention, how it was integrated, and any human‐AI interaction protocols

If the study is a clinical trial evaluating an AI intervention (e.g., comparing AI‐assisted diagnosis to standard care), provide a detailed description of the AI intervention itself. This includes how the AI system was integrated into the clinical workflow, who used it (e.g., surgeons, radiologists and nurses), what training they received, what information the AI provided, and how clinicians were expected to use or interact with that information (e.g., as a decision aid, for double reading, to override). This is crucial for understanding the intervention being tested [6].

### Part 3G: AI model evaluation

This section details how the performance of the developed or validated AI model was assessed.

#### Part 3G.i: Performance metrics used. Justification for chosen metrics

Authors must clearly specify all performance metrics used to evaluate the AI model and provide a justification for their choice, particularly in relation to the specific orthopaedic task and the clinical implications of different types of errors.

- For classification/diagnostic models, common metrics include accuracy, sensitivity (recall), specificity, positive predictive value (PPV/precision), negative predictive value (NPV), F1‐score, Matthews Correlation Coefficient (MCC) and area under the receiver operating characteristic curve (AUC‐ROC). The F1‐Score and MCC are particularly important for imbalanced datasets.
- For prognostic models, metrics include discrimination (e.g., AUC‐ROC, often C‐statistic for time‐to‐event data like implant survival) and calibration (e.g., calibration plots, Brier score) which assesses the agreement between predicted probabilities and observed outcomes. For the prediction of continuous outcomes mean average error, mean squared error and root mean squared error should be considered.
- For segmentation models, common metrics include Dice similarity coefficient (DSC), Intersection over Union (IoU or Jaccard index), Hausdorff distance, and average surface distance.

The choice of metrics should reflect the clinical priorities (e.g., is it more important to avoid false negatives or false positives for a specific orthopaedic condition?).

#### Part 3G.ii: Confidence intervals for all metrics

For all reported performance metrics, provide corresponding confidence intervals (CIs), typically 95% CIs. CIs quantify the uncertainty in the estimate of model performance due to sampling variability. Reporting CIs is crucial for interpreting the reliability and precision of the performance measures and for comparing across studies or models. The method used to calculate CIs (e.g., bootstrapping, exact methods) should also be stated.

#### Part 3G.iii: Details of internal and external validation (if performed), including description of the validation cohorts

Describe any methods used for internal validation (assessing performance on data from the same underlying population as the training data, e.g., using a hold‐out test set, cross‐validation, or bootstrapping) and, importantly, any external validation (assessing performance on entirely separate data, e.g., from different institutions, time periods, geographical locations, or with slightly different patient characteristics or imaging equipment). For external validation, provide a detailed description of the validation cohort(s), including their source, eligibility criteria, baseline characteristics, and how they differ from the development cohort. Robust external validation is critical for demonstrating a model′s generalisability and readiness for broader application.

#### Part 3G.iv: Error analysis: Investigation of false positives/negatives

Beyond aggregate performance metrics, authors should report on an analysis of the AI model′s errors (false positives and false negatives). This involves qualitatively and, if possible, quantitatively describing the characteristics of cases where the model failed. This could mean reviewing misclassified X‐rays to see if there are common features (e.g., subtle fractures, unusual anatomy, presence of implants, poor image quality) or examining patient profiles for incorrect prognostic predictions. Understanding why a model fails is crucial for identifying its limitations, potential biases, and areas for future improvement.

#### Part 3G.v: Comparison with existing methods or clinician performance (if applicable)

If a key objective is to assess whether the AI model performs comparably to, or better than, existing methods (e.g., traditional statistical models, simpler scoring systems) or human experts (e.g., orthopaedic surgeons, radiologists of varying experience levels), describe the comparative analysis. This includes how the comparator method/persons were chosen, how their performance was assessed (using the same metrics and reference standard as the AI model), and the statistical methods used for comparison.

### Part 3H: Explainability/interpretability

For complex AI models, particularly deep learning “black boxes,” it is increasingly important to report any methods used to understand or explain how the model arrives at its predictions. This is often referred to as eXplainable AI (XAI). Techniques might include generating saliency maps or heatmaps (e.g., Grad‐CAM, SHAP) that highlight which parts of an image were most influential for a decision, calculating feature importance scores (e.g., from tree‐based models or permutation importance), or using LIME (Local Interpretable Model‐agnostic Explanations). Reporting on explainability can enhance trust in the model and provide insights into its decision‐making process, potentially revealing whether it is learning clinically relevant features or relying on spurious correlations.

### Part 3I: Statistical methods

#### Part 3I.i: Methods for calculating sample size (if applicable)

If a formal sample size calculation was performed, such as for a prospective validation study or a clinical trial comparing AI to standard care, describe in detail the methodology used, including all assumptions (e.g., expected effect size, desired confidence interval width for a key performance metric, anticipated outcome rate, significance level, and statistical power). For many AI model development studies, especially retrospective ones, sample size is typically determined by data availability, but it remains important to justify and assess whether the sample size is adequate to achieve sufficient precision for key performance metrics. For prospective validation studies and clinical trials, sample size calculations should focus on achieving precise estimates, for example, by targeting narrow CIs, rather than relying solely on traditional hypothesis‐testing power calculations. Whenever possible, consider precision‐based sample size calculations and specify the targeted level of statistical certainty for the performance metrics reported.

#### Part 3I.ii: Methods for handling continuous variables

Describe how continuous predictor variables were handled in the model development process. Were they kept continuous (often preferred), or were they categorised or transformed (e.g., log transformation, polynomial terms)? If categorised, provide the cut‐points and rationale. The handling of continuous variables can significantly affect model performance and interpretability.

#### Part 3I.iii: Methods for handling missing data in model development and validation

This reiterates Part 3E.iii but focuses on the statistical methods employed specifically during model development and validation phases (as opposed to initial data sourcing). If missing data were imputed, provide details of the imputation model, the number of imputations if multiple imputation was used, and how imputation was handled in relation to data partitioning (e.g., imputation parameters derived only from the training set).

## IV. RESULTS

The section should present the findings of the study in a logical sequence, clearly and objectively, without interpretation or discussion (which belongs in the Discussion section). It should directly address the study′s objectives as stated in the Introduction.

### Part 4A: Participant/data flow

A flow diagram is useful for transparently reporting the number of participants or data units (e.g., images, patient records) at each stage of the study. This includes the initial number of potentially eligible individuals/data units, the number assessed for eligibility, the reasons for exclusion at each stage, and the final numbers included in the main analysis, as well as in different datasets (training, validation, testing). This diagram helps readers understand the selection process and potential for selection bias. For AI studies, it should clearly delineate the flow into development and different validation cohorts.

### Part 4B: Baseline characteristics: Demographics, clinical characteristics, and orthopaedic specifics of the development and validation cohorts

Present the baseline demographic, clinical, and relevant orthopaedic‐specific characteristics of the participants or data units included in the study, particularly for the development (training/validation) and test cohorts. This information is often best presented in a table. Characteristics should include those relevant to the orthopaedic condition, the AI model, and the outcome (e.g., age, sex, BMI, disease severity, implant types and baseline PROM scores). This allows readers to assess the similarity of the cohorts (important for interpreting internal validation) and the generalisability of the findings to their own populations. For external validation studies, clearly compare the characteristics of the development and external validation cohort(s).

### Part 4C: Model performance

#### Part 4C.i: Full performance metrics on all datasets as described in 3.G

Report all pre‐specified performance metrics (with their confidence intervals) for the AI model on the training, validation (if separate from training for final model selection), and, most importantly, the test set(s) (internal and any external). Performance on the training set can indicate overfitting if it is substantially higher than on the test set. Performance on independent test sets is the most crucial indicator of the model′s generalisability. Present these results clearly, often in tables.

#### Part 4C.ii: Calibration plots for prediction models

For AI models that output probabilities (e.g., risk of non‐union, likelihood of PJI), it is essential to report on their calibration. Calibration refers to how well the predicted probabilities agree with the observed frequencies of the outcome. A calibration plot (graphing predicted probabilities against observed frequencies across deciles or other groups of risk) is the one way to visualise this. Quantitative measures of calibration (e.g., Brier score, calibration slope and intercept) can alternatively be reported. A model can have good discrimination (e.g., high AUC) but poor calibration, which would make its probability outputs unreliable for clinical decision‐making.

### Part 4D: Error analysis insights

Summarise the findings from the error analysis (described in Methods Part 3G.iv). Present the patterns or characteristics observed in the false positive and false negative cases. Provide a clinical interpretation in the discussion of these errors where possible. This gives a more nuanced understanding of the model′s behaviour beyond summary statistics.

### Part 4E: Comparative performance: Results of AI versus human experts or other methods

If the study included a comparison of the AI model′s performance against human experts or existing methods, present the comparative results clearly. This should include the performance metrics (with CIs) for both the AI and the comparator(s) on the same dataset, and the statistical significance of any differences if tested.

### Part 4F: Visual examples of AI output

For many orthopaedic AI applications, particularly those involving imaging, providing visual examples of the AI model′s output can significantly enhance understanding and interpretation. This might include:

- Examples of correctly and incorrectly classified images (e.g., X‐rays with/without fractures, with the AI′s classification and any saliency maps).
- Segmentation overlays on images (e.g., AI‐drawn cartilage contours overlaid on an MRI, compared to expert contours)
- Heatmaps from explainability methods showing which image regions influenced the AI′s decision.

These visuals can make the AI's performance more tangible and help readers appreciate its strengths and weaknesses in specific orthopaedic contexts.

## V. DISCUSSION

The section is where authors interpret their findings in the context of existing knowledge, discuss the clinical and research implications, acknowledge limitations, and suggest future directions. It should not simply repeat the results.

### Part 5A: Statement of principal findings: In the context of orthopaedic care

Begin the discussion by clearly summarising the main findings of the study, directly relating them back to the study objectives and the specific orthopaedic problem being addressed. Avoid overstating the findings. Emphasise what the study adds to the understanding or application of AI in orthopaedic care.

### Part 5B: Clinical and research implications: How the findings could impact orthopaedic practice, patient care or future research

Discuss the potential implications of the study′s findings for orthopaedic clinical practice, patient care, or future research avenues. How could this AI model, if further developed or implemented, change how orthopaedic conditions are diagnosed, managed, or understood? What new research questions arise from these findings? Be realistic and avoid speculation not supported by the data.

### Part 5C: Limitations: Including biases, generalisability to different orthopaedic populations, settings, equipment or implant types

This is a critical part of the Discussion. Authors must provide a thoughtful and honest discussion of the study′s limitations. This includes:

- Potential sources of bias (e.g., selection bias due to retrospective design, spectrum bias if the study population was not representative, information bias).
- Limitations related to the data used (e.g., single‐center data, specific imaging protocols, limited sample size for certain subgroups).
- Issues affecting the generalisability of the AI model to different orthopaedic patient populations (e.g., different age groups, ethnicities, comorbidities), clinical settings (e.g., community vs. academic, different countries), imaging equipment (e.g., different MRI scanner strengths or X‐ray vendors not included in training), or variations in orthopaedic implants or surgical techniques.
- Limitations of the AI methodology itself (e.g., 'black box' nature if not addressed by XAI, specific architectural choices).

### Part 5D: Comparison with existing literature: Strengths and weaknesses relative to other AI models or conventional approaches in orthopaedics

Place the study′s findings within the context of existing published literature. Compare the AI model′s performance, methodology, and findings with those of other relevant AI models developed for similar orthopaedic tasks or with conventional (non‐AI) approaches. Discuss the strengths and weaknesses of the current study and its AI model in relation to prior work. This helps to position the study′s contribution and identify advancements or persistent challenges.

### Part 5E: Future Directions: Further development, validation or implementation steps

Conclude the discussion by outlining specific and actionable future directions. This could include plans for further model development (e.g., incorporating new data types, refining the architecture), necessary next steps for validation (e.g., prospective multicenter external validation, impact analysis studies), or research needed to explore the clinical implementation and real‐world utility of the AI system (e.g., workflow integration studies, health economic analyses and comparative effectiveness trials).

## DISCUSSION

The STORM‐AI checklist and this accompanying Explanation and Elaboration (E&E) document have been developed by the ESSKA AI Working Group to address the specific reporting needs of AI studies within our specialty. Our aim is to provide a practical framework that enhances the completeness and clarity of manuscripts, thereby enabling robust peer review, facilitating meta‐analyses, and ultimately fostering greater trust and utility in AI‐driven orthopaedic innovations.

The development of STORM‐AI was predicated on the understanding that while general AI reporting guidelines like CONSORT‐AI, STARD‐AI and TRIPOD offer a foundation, they do not fully capture the unique nuances inherent in orthopaedic research. STORM‐AI uniquely seeks to bridge this gap by incorporating orthopaedic‐specific considerations into a comprehensive reporting structure. For instance Part 3E.iv (Radiographic measurements, segmentation inputs, specific PROMs) directly addresses this need for domain‐specific detail, which is crucial for interpreting model performance and assessing generalisability across different scenarios.

The need for such guidelines is underscored by recent observations in the broader medical and orthopaedic literature. Concerns about the 'black box' nature of some AI models, the potential for algorithmic bias, and the challenges in external validation are frequently discussed [1, 9]. By promoting detailed reporting of methods (Section III), including data sources, model development, evaluation strategies, and explainability efforts (Part 3H), STORM‐AI aims to mitigate these concerns. The emphasis on clear descriptions of participant data flow (Part 4A), baseline characteristics (Part 4B), and error analysis (Part 4D) further contributes to this goal.

The adoption of STORM‐AI by authors and its endorsement by orthopaedic journals, has the potential to significantly elevate the quality of orthopaedic surgery related AI research. For authors, STORM‐AI serves as a roadmap for designing and reporting their studies comprehensively. For peer reviewers and editors, it provides a structured framework for critically appraising manuscripts, ensuring that key methodological details are present and adequately described. For readers and clinicians, transparently reported studies allow for a better understanding of an AI model′s capabilities, its limitations (Part 5C), and its potential utility in their own practice or research (Part 5B). This is particularly important as AI tools begin to transition from research settings to clinical decision support. Practical guides on AI implementation are emerging, but standardised reporting is a foundational step [7, 8, 10, 14, 15, 16].

The authors acknowledge several limitations of this work. Developed by a single society, the ESSKA AI Working Group, the guideline may reflect a predominantly European perspective and, given the rapid evolution of artificial intelligence, it should be considered a “living document” requiring periodic updates to remain current. Furthermore, the practical utility and impact of the STORM‐AI checklist have not yet been empirically validated through retrospective or prospective application, which represents a crucial next step. Finally, while comprehensive, this framework may not fully address the nuances of highly specialised or emerging AI subfields that may necessitate future extensions.

Future efforts may also focus on developing educational materials and workshops to facilitate the uptake and correct application of STORM‐AI.

## CONCLUSION

The STORM‐AI guidelines, developed by the ESSKA AI Working Group, offer a specialised framework for reporting AI research in orthopaedics. We believe that widespread adoption of these guidelines by authors, and endorsement by journals, will be instrumental in fostering robust, reliable, and clinically translatable AI innovations that ultimately benefit orthopaedic patients.

## AUTHOR CONTRIBUTIONS

All listed authors have contributed substantially to this work: Felix C. Oettl, Bálint Zsidai, Yinan Yu and James Pruneski performed literature review, and primary manuscript preparation. Editing and final manuscript preparation was performed by Thomas Tischer, Ayoosh Pareek, Alberto Grassi, Stefano Zaffagnini, Michael T. Hirschmann, and Kristian Samuelsson. All authors read and approved the final manuscript.

## CONFLICT OF INTEREST STATEMENT

Alberto Grassi: Smith & Nephew; not paid consultant. Stefano Zaffagnini: DePuy, A Johnson & Johnson Company: Paid presenter or speaker, paid consultant; European Society of Sports Traumatology Knee Surgery and Arthroscopy (ESSKA): Board or committee member; International Society of Arthroscopy, Knee Surgery, and Orthopaedic Sports Medicine (ISAKOS): Board or committee member; Journal of Experimental Orthopaedics (JEO): Editorial or governing board; Smith & Nephew: Paid presenter or speaker, paid consultant. Michael T Hirschmann is an editorial board member of KSSTA. Kristian Samuelson is a member of the Board of Directors of Getinge AB (publ) and medtech advisor to Carl Bennet AB.

## ETHICS STATEMENT

The authors have no ethics statement to report.

## Supporting information

STORM‐AI guideline Examples.

ESSKA STORM‐AI Checklist.

## Data Availability

Data sharing not applicable to this article as no datasets were generated or analysed during the current study.
