# Supplementary material for: Standardised Transparent Orthopaedic Reporting and Modelling for AI (STORM‐AI)—Guidelines for reporting artificial intelligence studies in orthopaedics from the ESSKA AI Working Group
Source: J Exp Orthop. 2026 Mar 30;13(2):e70702. doi: 10.1002/jeo2.70702 (PMC13052261; doi:10.1002/jeo2.70702)
Supplement: Supplementary file 1 — STORM‐AI guideline Examples. [file JEO2-13-e70702-s001.docx]

STORM-AI guideline Examples

All examples but the Titles were produced utilizing Gemini 2.5 Pro Preview 05-06 and reviewed by the ESSKA AI working group to be in line with our guidelines. Any likelihood with published works is purely confidential and should not be seen as endorsement for specific publications.

## Part 1a: Identification as a study of an AI model in orthopedics.

### Orthopedic Examples:

- "Deep Learning Model for Automatic Identification and Classification of Distal Radius Fracture.”
- "Development of Explainable Machine Learning Models to Predict Outcomes following Platelet-Rich Plasma Injections for Knee Osteoarthritis." (published soon)
- " Machine learning algorithm to predict anterior cruciate ligament revision demonstrates external validity."

## Part 1b: Specific orthopedic problem/application addressed.

### Orthopedic Examples: (Often integrated with Part 1a)

- "...for Automated Detection of *Occult* Scaphoid Fractures on Plain Radiographs to *Reduce Diagnostic Delays*."
- "...for Predicting *Risk of Revision Surgery due to Aseptic Loosening within 5 Years* after Total Hip Arthroplasty."
- "...for Differentiating *Benign from Malignant Primary Bone Tumors* on MRI using Texture Analysis and Machine Learning."

## Part 1c: Study objectives (e.g., developing, validating, or comparing an AI model).

### Orthopedic Examples (typically found in the Abstract):

- "Objective: To develop and internally validate a machine learning model to predict the likelihood of achieving a minimal clinically important difference (MCID) in the Oxford Knee Score 6 months following unicompartmental knee arthroplasty."
- "Objective: To externally validate a previously developed deep learning algorithm for the automated measurement of hip-knee-ankle angle on full-limb radiographs in an independent patient cohort from three different orthopedic centers."
- "Objective: To compare the diagnostic performance of a novel AI-based software with that of fellowship-trained orthopedic trauma surgeons for identifying subtle Lisfranc injuries on CT scans."

## Part 1d: Mention of model type (e.g., diagnostic, prognostic, segmentation).

### Orthopedic Examples (typically found in the Abstract):

- "We describe the development of a *convolutional neural network (CNN)-based diagnostic model* for identifying anterior cruciate ligament tears on MRI scans."
- "This study evaluates the performance of a *prognostic survival model incorporating machine learning techniques (e.g., random forest)* to predict long-term implant survivorship after total ankle replacement."
- "The *AI-powered image segmentation tool* utilized a modified U-Net architecture to automatically quantify muscle fatty infiltration in patients with rotator cuff pathology."

# II. INTRODUCTION

## Part 2a: Background & Objectives - Scientific and clinical orthopedic background, including the intended use of the AI.

### Orthopedic Examples:

- "Osteoarthritis (OA) of the knee is a leading cause of disability worldwide, and accurate assessment of its severity from plain radiographs, typically using the Kellgren-Lawrence (KL) grading system, is essential for clinical management and research. However, manual KL grading suffers from significant intra- and inter-observer variability. An automated AI system for consistent and accurate KL grading could standardize OA severity assessment, improve clinical trial efficiency, and support clinical decision-making. This study aims to develop and validate such an AI system for use in outpatient orthopedic clinics and large-scale epidemiological research."
- "Periprosthetic joint infection (PJI) following total hip arthroplasty (THA) is a serious complication associated with high morbidity and healthcare costs. Early and accurate diagnosis is critical but can be challenging due to the limitations of current diagnostic tests, including serum markers and synovial fluid analysis, which may be inconclusive or require invasive procedures. An AI model capable of accurately predicting PJI risk based on readily available pre-operative and early post-operative patient data could identify high-risk individuals for targeted interventions or intensified surveillance. The intended use is as a clinical decision support tool for orthopedic surgeons managing THA patients."

## Part 2b: Background & Objectives - Specific objectives and hypotheses, clearly stating if it's development, validation, or both.

## Orthopedic Examples:

- "The primary objective of this study was to develop and internally validate an AI model using deep learning techniques to automatically segment the femoral head and acetabulum on anteroposterior pelvic radiographs for the purpose of calculating radiographic hip parameters relevant to femoroacetabular impingement syndrome."
- "Our specific aims were: (1) To externally validate a previously published AI algorithm for predicting non-union after tibial shaft fracture fixation using data from our institutional trauma registry, and (2) To assess its calibration performance and clinical utility in our patient population. We hypothesized that the model's discrimination would be maintained in our cohort."
- "The objective of this prospective study was to compare the accuracy of a novel AI-based system for detecting subtle meniscal tears on knee MRI scans against the consensus interpretation of two fellowship-trained musculoskeletal radiologists, with arthroscopic findings serving as the reference standard."

## Part 2c: Background & Objectives - Intended population and setting (e.g., primary TKA patients in a tertiary center).

### Orthopedic Examples:

- "The intended population for this diagnostic AI tool is adult patients presenting to outpatient orthopedic clinics or emergency departments with suspected acute ankle fractures, prior to definitive radiographic interpretation by a radiologist."
- "This prognostic AI model is designed for pre-operative risk stratification of adult patients scheduled for elective primary total knee arthroplasty due to end-stage osteoarthritis, within the context of a high-volume, specialized arthroplasty unit."
- "The AI-driven segmentation software is intended for research applications analyzing knee MRI scans from large-scale longitudinal cohort studies investigating risk factors and progression markers of knee osteoarthritis in a population aged 45-79 years."

# III. METHODS

## Part 3A: Study Design

## Part 3A.i: Prospective or retrospective.

### Orthopedic Examples:

- "This was a retrospective cohort study utilizing electronic health records and radiographic data collected between January 2015 and December 2020."
- "We conducted a prospective diagnostic accuracy study where patients undergoing knee MRI for suspected meniscal tears were consecutively enrolled, and the AI model's assessment was compared to subsequent arthroscopic findings."
- "The AI model was developed using retrospective data and then prospectively validated in a time-split manner using all eligible patients presenting from January 2021 to June 2022."

## Part 3A.ii: Description of the study population and period.

### Orthopedic Examples:

- "The study population included all adult patients who underwent primary, elective total knee arthroplasty for osteoarthritis at our institution between June 1, 2018, and May 31, 2022."
- "Data were sourced from a multicenter trauma registry, encompassing all patients aged 16 years or older presenting with tibial plateau fractures between January 2017 and December 2021."

## Part 3A.iii: Setting (e.g., multicenter, single academic hospital, public registry).

### Orthopedic Examples:

- "This was a single-center study conducted at a university-affiliated tertiary care orthopedic hospital in North America."
- "Data were obtained from three affiliated community hospitals and one academic medical center, all part of the same regional health network."
- "The study utilized data from the National Joint Registry for England, Wales, Northern Ireland and the Isle of Man, a public database of arthroplasty procedures."

## Part 3B: Participants/Data Source

## Part 3B.i: Eligibility criteria (inclusion/exclusion) for patients/data.

### Orthopedic Examples:

- "Inclusion criteria were: (1) patients aged 18 years or older; (2) undergoing primary cementless total hip arthroplasty for osteoarthritis; (3) availability of preoperative anteroposterior pelvic radiographs and postoperative radiographs at 1-year follow-up. Exclusion criteria were: (1) history of prior hip surgery on the ipsilateral side; (2) developmental hip dysplasia; (3) radiographic images of insufficient quality for AI analysis as determined by two orthopedic surgeons."
- "We included all shoulder MRI studies performed between [date] and [date] on patients aged 16-80 years with suspected rotator cuff pathology. Studies were excluded if they had significant motion artifact, were post-operative, or if the imaging protocol did not include standard sagittal T2-weighted and coronal oblique proton-density weighted sequences."

## Part 3B.ii: Source of data (e.g., PACS, EHR, orthopedic registries, wearable sensors).

### Orthopedic Examples:

- "Preoperative radiographs were retrieved from the institutional PACS, and clinical demographic data, operative details, and patient-reported outcome measures (PROMs) were extracted from our orthopedic department's prospectively maintained arthroplasty registry, which is linked to the hospital's EHR."
- "Gait data were collected using three inertial measurement unit (IMU) sensors (Model X, Manufacturer Y) attached to the lumbar spine and bilateral tibiae of participants during a 10-meter walk test."
- "Data on implant survivorship and reasons for revision were obtained from the [National/Regional Orthopedic Registry]."

## Part 3B.iii: Methods of data collection and any pre-processing specific to orthopedic data (e.g., image standardization for radiographs/MRI, handling of implant artifacts, extraction from operative notes).

### Orthopedic Examples:

- "All knee radiographs were obtained using a standardized protocol (weight-bearing, fixed flexion, specific source-to-image distance). Images were pre-processed by converting DICOM files to PNG format, followed by intensity normalization to a range of. Regions of interest (ROIs) centered on the knee joint were automatically cropped to a size of 512x512 pixels."
- "For MRIs, a specific protocol including sagittal T1-weighted, axial T2-weighted fat-suppressed, and coronal STIR sequences was used. Metal artifact reduction sequences (MARS) were employed for patients with existing hardware if feasible. All images were visually inspected for quality by a research fellow."
- "Operative notes in free-text format were processed using a custom NLP pipeline based on regular expressions and a predefined dictionary of orthopedic terms to extract implant type, fixation method, and reported intraoperative complications."
- "Fracture classifications (e.g., AO/OTA) were assigned to each case by two fellowship-trained orthopedic trauma surgeons based on CT scans, with disagreements resolved by a third senior surgeon. These classifications served as input features for the prognostic model."

## Part 3C: Outcome Definition

## Part 3C.i: Clear definition of the outcome(s) being predicted (e.g., implant survivorship, non-union, specific PROM threshold, post-operative complication).

### Orthopedic Examples:

- "The primary outcome was all-cause revision surgery of the index total knee arthroplasty, defined as any subsequent surgical procedure resulting in removal or exchange of one or more components of the prosthetic joint, as recorded in the institutional arthroplasty registry."
- “Non-union of a tibial shaft fracture was defined at 9 months post-operatively based on the FDA criteria: radiographic evidence of a fracture line with no bridging callus formation over three of four cortices on orthogonal X-ray views, and clinical symptoms including pain on weight-bearing or motion at the fracture site, requiring secondary intervention."
- "A 'good clinical outcome' following ACL reconstruction was defined as achieving a Patient Acceptable Symptom State (PASS) threshold on the Knee injury and Osteoarthritis Outcome Score (KOOS) Quality of Life subscale (PASS threshold ≥ 62.5 points) at 12 months post-surgery."
- "Surgical site infection (SSI) was defined according to the Centers for Disease Control and Prevention (CDC) criteria for superficial or deep incisional SSI occurring within 90 days of the index orthopedic procedure."
- "The presence of a scaphoid fracture was defined by confirmation on CT scan or MRI performed within 2 weeks of the initial wrist X-ray, or by union visible on follow-up radiographs at 6 weeks if no advanced imaging was performed."

## Part 3C.ii: How and when outcomes were assessed, including follow-up duration.

### Orthopedic Examples:

- "Outcome data on implant revision were collected through annual linkage with the National Joint Registry, with a minimum follow-up of 5 years and a maximum of 15 years for all patients included in the development cohort."
- "Diagnosis of periprosthetic shoulder infection was made according to the 2018 International Consensus Meeting (ICM) criteria, assessed by a multidisciplinary team of orthopedic surgeons and infectious disease specialists at the time of any re-operation or specific diagnostic workup during the first 2 years post-arthroplasty."
- "Patient-reported outcome measures (IKDC, KOOS) were collected electronically via an online platform at baseline, 6 months, 12 months, and 24 months post-operatively. Trained research staff contacted non-responders via telephone."
- "The presence or absence of rotator cuff tear on MRI (the outcome for the AI diagnostic model) was determined by the consensus reading of two fellowship-trained musculoskeletal radiologists with over 10 years of experience each, who were blinded to the AI model's output and clinical information other than patient age and sex."

## Part 3C.iii: Any cut-offs used for continuous outcomes.

### Orthopedic Examples:

- "The Western Ontario and McMaster Universities Osteoarthritis Index (WOMAC) pain score, ranging from 0 to 20, was dichotomized to define a 'responder' as a patient achieving a score ≤ 5 at 6 months post-TKA, based on previously established patient acceptable symptom state values for this population."
- "Radiographic progression of osteoarthritis was defined as a decrease of ≥ 0.5mm in medial joint space width on serial weight-bearing knee radiographs over 2 years. This cut-off was chosen based on its use in previous large OA trials."
- "For predicting 'high' versus 'low' bone mineral density (BMD), the T-score from DXA scans was categorized using the World Health Organization (WHO) thresholds (T-score ≤ -2.5 for osteoporosis, -2.5 < T-score < -1.0 for osteopenia, and T-score ≥ -1.0 for normal BMD)."

## Part 3D: Reference Standard (Primarily STARD - for diagnostic/detection models)

## Part 3D.i: The "ground truth" used to establish the presence/absence or extent of the orthopedic condition (e.g., arthroscopic findings for cartilage lesions, histology for tumors, expert panel consensus for fracture detection on X-rays).

### Orthopedic Examples:

- "The reference standard for the presence and grade of cartilage lesions in the knee was direct visualization and probing during arthroscopic surgery, documented on standardized diagrams by the operating surgeon."
- "Histopathological analysis of biopsy specimens by an experienced musculoskeletal pathologist served as the reference standard for differentiating benign from malignant bone tumors."
- "For the detection of occult scaphoid fractures on initial radiographs, the reference standard was the presence of a fracture line on a CT scan performed within 10 days of injury or, in cases where CT was not performed, clear evidence of fracture healing (bridging callus) on follow-up radiographs at 6-8 weeks."
- "The reference standard for classifying rotator cuff tear severity on MRI was the consensus reading of three fellowship-trained musculoskeletal radiologists, each with over 10 years of experience, who independently reviewed the images and then resolved discrepancies through discussion."

## Part 3D.ii: Rationale for choosing the reference standard.

### Orthopedic Examples:

- "Arthroscopy was chosen as the reference standard for meniscal tears as it is the current gold standard for direct visualization and confirmation of such pathology, allowing for tactile feedback through probing."
- "Although long-term clinical follow-up can confirm scaphoid fractures, CT imaging was chosen as the primary reference standard due to its high sensitivity and specificity for acute bony injury and its ability to provide confirmation within a clinically actionable timeframe for the study."
- "Expert consensus panel reading was selected as the reference standard for radiographic osteoarthritis grading because, while no perfect gold standard exists, consensus among experienced readers is a commonly accepted method to minimize individual reader variability and establish a robust benchmark in imaging research."

## Item 3D.iii: Blinding of assessors to AI results and other clinical information.

### Orthopedic Examples:

- "The orthopedic surgeons performing arthroscopy (reference standard) were blinded to the AI model's interpretation of the pre-operative MRI scans."
- "Radiologists interpreting the CT scans (reference standard for fracture detection) were blinded to the AI model's output on the initial plain radiographs and to all clinical information except for patient age and anatomical site imaged."
- "The orthopedic residents whose radiographic interpretations were compared to the AI model were blinded to the AI model's findings and the final reference standard diagnosis."

## Part 3E: Predictors/Input Data

## Part 3E.i: Detailed description of all input data used by the AI (e.g., demographic, clinical, imaging features, intraoperative data, biomechanical parameters).

### Orthopedic Examples:

- Prognostic Model for TKA Outcome: "Input variables for the prediction model included: (1) Demographics: age at surgery, sex, body mass index (BMI); (2) Clinical: baseline Oxford Knee Score, ASA grade, presence of diabetes, smoking status; (3) Radiographic: preoperative Kellgren-Lawrence grade, hip-knee-ankle angle; (4) Intraoperative: femoral and tibial component sizes, tourniquet time."
- Diagnostic Model for Fracture Detection: "The AI model used raw pixel data from anteroposterior and lateral view DICOM images of the wrist as input. No manual feature extraction was performed."
- Segmentation Model for Cartilage: "Input to the U-Net model consisted of 2D slices from sagittal T2-weighted fat-suppressed knee MRI sequences, specifically a 256x256 pixel patch centered on the medial femoral condyle."
- Radiomics Model for Tumor Classification: "A total of 107 radiomic features (first-order statistics, shape features, texture features including GLCM, GLRLM, GLSZM, NGTDM, and GLDM) were extracted from manually segmented tumor regions on T1-weighted post-contrast MRI sequences."

## Part 3E.ii: How and when these were measured/extracted.

### Orthopedic Examples:

- "Preoperative WOMAC scores were collected within 4 weeks prior to surgery using a standardized electronic questionnaire administered in the outpatient clinic."
- "Femoral anteversion and neck-shaft angle were measured on preoperative CT scans using a validated semi-automated 3D reconstruction software (Software X, Version Y) by a trained research assistant."
- "Comorbidities were extracted from the hospital's EHR using ICD-10 codes recorded within 12 months prior to the index admission."
- "Intraoperative ligament balance data (varus/valgus laxity in degrees at 0°, 30°, 60°, and 90° of flexion) were recorded prospectively using a sensor-integrated tibial trial component (Device Z) by the operating surgeon after final implant positioning but before wound closure."

## Part 3E.iii: Handling of missing data.

### Orthopedic Examples:

- "For the 7% of patients with missing preoperative BMI data, multiple imputation using chained equations (5 imputations) was performed based on age, sex, and baseline functional score."
- "Patients with any missing predictor variables required for the model (n=32, 4.5% of initial cohort) were excluded from the analysis (complete case analysis)."
- "Missing values for radiographic measurements (e.g., joint space width, occurring in <2% of cases) were imputed using the median value from the training dataset. A missingness indicator variable was also created and included as a potential predictor."
- "The LightGBM algorithm used for model development inherently handles missing values by assigning them to the branch that maximizes information gain at each split."

## Part 3E.iv: Specific radiographic measurements used, details of segmentation (if input to another model), specific PROMs.

### Orthopedic Examples:

- "Radiographic inputs included: (1) Kellgren-Lawrence grade (0-4) assessed on weight-bearing AP knee radiographs by two orthopedic residents; (2) Medial and lateral tibial slope (degrees) measured on sagittal reconstructed CT images using the method described by Dejour et al.; (3) Alpha angle (degrees) measured on Dunn lateral hip radiographs."
- "The input to the prognostic model included the volumetric bone mineral density (vBMD) of the L1-L4 vertebrae, which was derived from an automated AI-driven segmentation and analysis of preoperative CT scans (for details of the segmentation AI, see [citation])."
- "Patient-reported outcome measures used as predictors included the Hip disability and Osteoarthritis Outcome Score (HOOS) subscales for Pain, Symptoms, Activities of Daily Living, Sport/Recreation, and Quality of Life, collected at baseline."

## Part 3F: AI Model Development & Training

## Part 3F.i: Description of the AI model architecture (e.g., CNN type, layers, decision tree, etc.).

### Orthopedic Examples:

- "We utilized a pre-trained ResNet-50 architecture, with the final fully connected layer replaced by a new layer with a sigmoid activation function for binary classification of fracture presence."
- "A U-Net based architecture with an EfficientNetB3 encoder backbone was used for semantic segmentation of the femoral cartilage. The decoder consisted of five up-sampling blocks with skip connections from the encoder."
- "A random forest classifier was developed, consisting of 500 decision trees. The Gini impurity was used to measure split quality, and the maximum depth of each tree was limited to 10 levels."
- "The prognostic model was a logistic regression model with L2 regularization (Ridge). Predictors were selected using a backward elimination approach based on Akaike Information Criterion (AIC)."

## Part 3F.ii: Data pre-processing steps not covered in III.B (e.g., data augmentation techniques relevant for orthopedic images).

### Orthopedic Examples:

- "During training of the CNN, on-the-fly data augmentation was applied to the input X-ray images, including random rotations (-15 to +15 degrees), horizontal flips (probability 0.5), random scaling (0.9x to 1.1x), and small Gaussian noise addition (sigma=0.01)."
- "All continuous clinical variables (e.g., BMI, age) were standardized by subtracting the mean and dividing by the standard deviation of the training set values before being input into the logistic regression model."
- "For the radiomics features, those with near-zero variance were removed. The remaining features were then normalized using z-score transformation."

## Part 3F.iii: Data partitioning: clear description of training, tuning/validation, and test sets (including sample sizes for each). Specify if test set was internal or external.

### Orthopedic Examples:

- "The dataset of 1200 knee radiographs was randomly partitioned at the patient level into a training set (n=840, 70%), a validation set (n=120, 10%), and an internal test set (n=240, 20%). Stratification by fracture presence was used to ensure similar proportions in each set."
- "The model was developed using data from Institution A (n=550 patients). Hyperparameter tuning was performed using 5-fold cross-validation on this development set. The final model was then tested on an external validation cohort of 230 patients from Institution B, which was collected independently."
- "A temporal split was used: data from patients undergoing surgery between 2015-2018 (n=780) were used for training and validation, and data from 2019-2020 (n=350) served as the unseen internal test set."

## Part 3F.iv: Details of the training process (e.g., loss function, optimization algorithm, hyperparameters, software/libraries used).

### Orthopedic Examples:

- "The CNN was trained for 100 epochs using the Adam optimizer with an initial learning rate of 0.001, a batch size of 32, and binary cross-entropy loss. Early stopping was implemented based on validation set performance (patience of 10 epochs). Training was performed using PyTorch (Version 1.9) on NVIDIA V100 GPUs."
- "The random forest model was trained using the randomForest package (Version 4.6-14) in R (Version 4.1.0). Hyperparameters (number of trees, mtry) were tuned using a grid search with 5-fold cross-validation, optimizing for the area under the ROC curve (AUC)."
- "For the U-Net segmentation model, a combined Dice loss and binary cross-entropy loss was used. The model was trained for 200 epochs with a learning rate of 1e-4, decayed by a factor of 0.1 when validation loss plateaued."

## Part 3F.v: Approach to model selection or feature selection.

### Orthopedic Examples:

- "Predictors for the logistic regression model were selected using a backward stepwise elimination approach based on the Akaike Information Criterion (AIC) in the training dataset."
- "For the radiomics analysis, a LASSO (Least Absolute Shrinkage and Selection Operator) logistic regression model was used for simultaneous feature selection and model construction, with the regularization parameter (lambda) chosen via 10-fold cross-validation to maximize AUC."
- "We trained three different CNN architectures (ResNet-34, ResNet-50, EfficientNet-B0) and selected the ResNet-50 based on its superior performance (Dice coefficient) on the internal validation set for cartilage segmentation."

## Part 3F.vi: For clinical trials: Description of the AI intervention, how it was integrated, and any human-AI interaction protocols.

### Orthopedic Examples:

- "In the intervention arm, orthopedic residents reviewed wrist radiographs with the assistance of the AI system, which provided an overlay highlighting suspected fracture locations and a probability score for fracture presence. Residents were instructed to consider the AI output but make their own final diagnostic decision."
- "The AI-driven surgical navigation system provided real-time guidance on implant positioning during TKA. Surgeons in the intervention group were trained on the system via a standardized didactic and simulation protocol and were instructed to follow the AI's recommendations for bone cuts and component alignment unless clinically contraindicated, in which case deviations and reasons were documented."

## Part 3G: AI Model Evaluation

## Part 3G.i: Performance metrics used (e.g., accuracy, F1-score, AUC-ROC/PR). Justification for chosen metrics.

### Orthopedic Examples:

- Fracture Detection (Classification): "Model performance was evaluated using sensitivity, specificity, positive predictive value (PPV), negative predictive value (NPV), accuracy, and AUC-ROC. Emphasis was placed on sensitivity due to the clinical importance of not missing a fracture."
- Predicting PJI (Prognostic): "The model's discriminative ability was assessed using the AUC-ROC. Calibration was evaluated using a calibration plot and the Brier score, as well-calibrated probabilities are crucial for clinical decision-making regarding PJI risk."
- Femoral Cartilage Segmentation: "Segmentation accuracy was quantified using the Dice similarity coefficient (DSC) to assess volumetric overlap, and the average symmetric surface distance (ASSD) to evaluate boundary concordance with the expert manual segmentations."

## Part 3G.ii: Confidence intervals for all metrics.

### Orthopedic Examples:

- "The sensitivity for detecting scaphoid fractures was 0.85 (95% CI, 0.78-0.91), and the specificity was 0.92 (95% CI, 0.88-0.95)."
- "The AUC-ROC for predicting 5-year implant survivorship was 0.78 (95% CI, 0.72-0.84), calculated using DeLong's method."
- "The mean Dice coefficient for femoral cartilage segmentation was 0.89 (95% CI, 0.87-0.91), with CIs estimated from 1000 bootstrap samples."

## Part 3G.iii: Details of internal and external validation (if performed), including description of the validation cohorts.

### Orthopedic Examples:

- Internal Validation: "Internal validation was performed using a randomly selected, held-out test set comprising 20% of the initial dataset, which was not used during model training or hyperparameter tuning. Performance was also assessed using 10-fold cross-validation within the training/validation partition."
- External Validation: "The model, developed using data from Hospital A, underwent external validation on two independent cohorts: Cohort 1 from Hospital B (a similar academic center in a different city, n=250 patients, data collected 2019-2021) and Cohort 2 from a public imaging database (OrthoImageDB, n=400 images from various scanners and international sites). Key differences in patient demographics (e.g., age distribution in Cohort 2) and imaging protocols (e.g., X-ray vendor in Cohort 1) are detailed in Table X."

## Part 3G.iv: Error analysis: Investigation of false positives/negatives with orthopedic context.

### Orthopedic Examples:

- "A review of 20 false negative cases for fracture detection revealed that 15 (75%) involved minimally displaced hairline fractures of the carpal bones, and 5 (25%) were associated with suboptimal radiographic positioning (e.g., excessive ulnar deviation). False positives (n=30) frequently occurred in cases with overlying soft tissue calcifications or accessory ossicles."
- "For the PJI prediction model, an analysis of false positives (patients predicted high risk but did not develop PJI) showed a higher prevalence of inflammatory comorbidities like rheumatoid arthritis. False negatives (predicted low risk but developed PJI) were more common in patients with delayed wound healing noted in their early postoperative records."

## Part 3G.v: Comparison with existing methods or clinician performance (if applicable).

### Orthopedic Examples:

- "The AI model's diagnostic accuracy for detecting meniscal tears on MRI was compared to that of three orthopedic residents (PGY3-5) and two fellowship-trained musculoskeletal radiologists. All human readers independently reviewed the same test set images, blinded to the AI results and arthroscopic findings. Their performance was evaluated using the same metrics (sensitivity, specificity, AUC) as the AI."
- "Performance of the AI-based prognostic score for 5-year THA survivorship was compared against a previously validated Cox proportional hazards model that used only demographic predictors. The comparison was based on AUC-ROC and Net Reclassification Improvement (NRI)."

## Part 3H: Explainability/Interpretability

## Part 3H.i: Methods used to understand or explain model predictions (e.g., saliency maps on X-rays, feature importance scores).

### Orthopedic Examples:

- "To understand the CNN's decision-making for fracture detection, Gradient-weighted Class Activation Mapping (Grad-CAM) was used to generate heatmaps overlaid on the input radiographs, highlighting regions most indicative of a fracture according to the model."
- "For the random forest model predicting non-union, feature importance was assessed using the mean decrease in Gini impurity and permutation importance scores for each input variable."
- "SHAP (SHapley Additive exPlanations) values were calculated to illustrate the contribution of each predictor to individual patient risk scores for PJI."

## Part 3I: Clinical Utility/Workflow

## Part 3I.i: How the AI system is intended to be used in the orthopedic clinical workflow.

### Orthopedic Examples:

- "The AI fracture detection tool is intended as a second reader or triage system for radiographs in the emergency department, flagging suspicious cases for urgent review by a radiologist or orthopedic specialist."
- "The prognostic model for TKA outcomes is designed to be used by surgeons during preoperative patient counseling to provide personalized risk/benefit information and aid in shared decision-making."
- "The AI-driven segmentation tool for muscle volume on MRI is intended for research use to quantify sarcopenia in orthopedic cohorts, not for direct clinical diagnosis by treating physicians."

## Part 3I.ii: Any human oversight or decision-making process involved.

### Orthopedic Examples:

- "The AI system provides a probability of malignancy for bone lesions; however, the final decision to biopsy or observe is made by the treating orthopedic oncologist, considering the AI output alongside all other clinical and imaging data."
- "While the AI system automatically segments and measures joint space width, all segmentations are reviewed and, if necessary, manually corrected by a trained research assistant before the measurements are used for analysis."
- "The AI for intraoperative surgical guidance provides recommendations for implant alignment, but the surgeon retains final control and can deviate based on intraoperative findings and clinical judgment, with any such deviations documented."

## Part 3J: Statistical Methods

## Part 3J.i: Methods for calculating sample size (if applicable).

### Orthopedic Examples:

- "A sample size of 200 patients in the test set was calculated to provide 80% power to detect a difference of at least 0.10 in AUC-ROC between the AI model and clinician performance, assuming an AUC of 0.75 for clinicians and a two-sided alpha of 0.05, based on a prior pilot study."
- "No formal sample size calculation was performed for this retrospective model development study; all available eligible cases from the 5-year period were included to maximize the training dataset size."

## Part 3J.ii: Methods for handling continuous variables.

### Orthopedic Examples:

- "Patient age and BMI were included as continuous linear terms in the logistic regression model."
- "Preoperative WOMAC pain score was initially considered as a continuous variable. However, due to a non-linear relationship with the outcome observed in exploratory plots, it was categorized into three groups (0-5, 6-10, 11-20) based on clinical relevance."
- "Tibial slope (degrees) was included as a continuous variable, and linearity was assessed using restricted cubic splines."

## Part 3J.iii: Methods for handling missing data in model development and validation.

### Orthopedic Examples:

- "Multiple imputation by chained equations (MICE) was performed separately within the training and test sets to prevent data leakage, generating 10 imputed datasets. Model development and evaluation were performed on each imputed dataset, and results were pooled according to Rubin's rules."
- "For variables with <5% missingness, median imputation based on the training set values was used prior to model fitting. Variables with >40% missing data were excluded from model consideration."

# IV. RESULTS

## Part 4A: Participant/Data Flow (Diagram showing flow of participants/data units through the study).

### Orthopedic Examples:

- "A flow diagram (Figure X) illustrates the patient selection process. Initially, 1578 patients undergoing primary TKA were identified. After applying exclusion criteria (e.g., revision surgery, incomplete records, specific comorbidities), 1250 patients remained. These were then split into development (n=900) and internal test (n=350) cohorts. For external validation, 450 eligible patients were identified from Hospital B, of whom 410 had complete data for analysis."
- "Figure X shows the data flow for image inclusion. From an initial pool of 5000 trauma radiographs, 800 were excluded due to poor image quality or incorrect labeling. The remaining 4200 images (from 3500 patients) were divided by patient into training (2800 images), validation (700 images), and test (700 images) sets."

## Part 4B: Baseline Characteristics: Demographics, clinical characteristics, and orthopedic specifics of the development and validation cohorts.

### Orthopedic Examples:

- "Table 1 presents the baseline characteristics of patients in the training and internal test sets for the PJI prediction model. There were no significant differences in age, sex, BMI, prevalence of diabetes, or ASA grade between the two sets. The median preoperative WOMAC score was X in the training set and Y in the test set."
- "The external validation cohort for the fracture detection AI (from Hospital B) had a significantly higher mean age (55 vs. 48 years, p=0.02) and a higher proportion of images from DR systems (80% vs. 60%, p<0.01) compared to the development cohort (Hospital A). See Table Y for full comparison."

## Part 4C: Model Performance

## Part 4C.i: Full performance metrics on all datasets as described in III.G.

### Orthopedic Examples:

- "On the internal test set (n=240), the AI model for fracture detection achieved an AUC-ROC of 0.92 (95% CI, 0.88-0.95), sensitivity of 0.88 (95% CI, 0.81-0.93), and specificity of 0.94 (95% CI, 0.90-0.97). Performance on the training set was AUC-ROC 0.99. On the external validation cohort from Hospital B (n=230), the AUC-ROC was 0.87 (95% CI, 0.81-0.92)."
- "The mean Dice coefficient for femoral cartilage segmentation on the test set was 0.89 (95% CI, 0.87-0.91), and the average symmetric surface distance was 0.45mm (95% CI, 0.41-0.49mm)."

## Part 4C.ii: Calibration plots for prediction models.

### Orthopedic Examples:

- "Figure 2 shows the calibration plot for the PJI prediction model on the external validation set, demonstrating good agreement between predicted probabilities and observed infection rates across deciles of risk. The calibration slope was 0.95 (ideal 1.0) and the intercept was -0.05 (ideal 0.0). The Brier score was 0.12."
- "The Hosmer-Lemeshow goodness-of-fit test indicated no significant lack of fit for the prognostic model (p=0.35 on the test set)."

## Part 4D: Error Analysis Insights: Clinical interpretation of common errors.

### Orthopedic Examples:

- "The error analysis of the fracture detection AI (Table X) showed that 60% of false negatives were hairline fractures in the carpal bones, particularly the scaphoid waist, often with minimal displacement. False positives were frequently triggered by prominent nutrient canals or old, healed fractures with residual sclerosis."
- "Review of the 15 cases where the AI model incorrectly predicted high risk of readmission (false positives) revealed that 10 (67%) had documented complex social situations or lived alone, factors not explicitly included in the model but potentially influencing actual readmission decisions by care teams."

## Part 4E: Comparative Performance: Results of AI vs. human experts or other methods.

### Orthopedic Examples:

- "The AI model achieved an AUC-ROC of 0.92 (95% CI, 0.88-0.95) for detecting meniscal tears, significantly higher than the mean AUC-ROC for orthopedic residents (0.78; 95% CI, 0.72-0.84; p=0.003) and comparable to fellowship-trained radiologists (0.90; 95% CI, 0.85-0.94; p=0.35). See Table Z for sensitivity/specificity comparisons."
- "Compared to a traditional logistic regression model using only demographic data (AUC 0.65), the AI model incorporating imaging features demonstrated superior discrimination for predicting 1-year poor functional outcome after ACL reconstruction (AUC 0.79, p<0.001 for difference in AUCs)."

## Part 4F: Visual examples of AI output (e.g., correctly/incorrectly classified images, segmentation overlays, heatmaps on orthopedic images).

### Orthopedic Examples:

- "Figure 3 displays representative examples of: (A) a correctly identified scaphoid fracture with the Grad-CAM overlay highlighting the fracture line; (B) a false negative case where a subtle fracture was missed by the AI; (C) a false positive where an accessory ossicle was misinterpreted as a fracture."
- "Figure 4 shows an example of the AI's automated segmentation of the femoral and tibial cartilage compartments on a knee MRI slice, alongside the manual reference segmentation, demonstrating good overall agreement but some underestimation in an area of severe cartilage thinning."

# V. DISCUSSION

## Part 5A: Statement of Principal Findings: In the context of orthopedic care.

### Orthopedic Examples:

- "In this study, we successfully developed and internally validated a deep learning model that demonstrated high accuracy for detecting distal radius fractures on plain radiographs, with performance comparable to orthopedic residents in a simulated setting. The model showed particular strength in identifying complete, displaced fractures."
- "Our external validation of a previously developed AI algorithm for predicting 90-day PJI risk after TKA showed moderate discrimination but excellent calibration in a new patient population, suggesting potential utility for risk stratification if further validated prospectively."

## Part 5B: Clinical & Research Implications: How the findings could impact orthopedic practice, patient care, or future research.

### Orthopedic Examples:

- "If prospectively validated, this AI tool for automated Kellgren-Lawrence grading could standardize osteoarthritis severity assessment in large clinical trials and potentially aid in identifying patients for earlier intervention in clinical practice. Future research should explore its sensitivity to change over time."
- "The ability of our AI model to predict patients at high risk for poor functional outcomes following ACL reconstruction using only preoperative data could enable surgeons to better counsel patients, manage expectations, and potentially tailor prehabilitation or surgical strategies. Further research is needed to determine if interventions based on these predictions can improve outcomes."

## Part 5C: Limitations: Including biases, generalizability to different orthopedic populations, settings, equipment, or implant types.

### Orthopedic Examples:

- "Our study has several limitations. First, its retrospective single-center design may limit generalizability; the AI model was trained on data from a single X-ray vendor, and its performance on images from other vendors is unknown. Second, while we performed internal validation, external validation in a different healthcare system is crucial before clinical deployment. Third, the model does not currently differentiate between acute and chronic fractures, which is a necessary refinement. Fourth, spectrum bias might be present as only patients who received a CT scan for confirmation were included in the reference standard for some fracture types."
- "A limitation of our PJI prediction model is its reliance on data from a high-volume arthroplasty center; its performance in lower-volume settings or with different patient demographics needs investigation. Furthermore, we did not include intraoperative variables like surgical duration, which could be important predictors."

## Part 5D: Comparison with Existing Literature: Strengths and weaknesses relative to other AI models or conventional approaches in orthopedics.

### Orthopedic Examples:

- "The AUC of 0.85 achieved by our model for predicting 1-year mortality after hip fracture surgery is comparable to the 0.82 reported by Smith et al. using a different machine learning algorithm but higher than the 0.75 achieved by a traditional regression model in the same study. Our model has the advantage of incorporating mobility data from wearable sensors, which was not used previously."
- "Unlike previous AI models for knee OA grading that relied on manually drawn regions of interest, our end-to-end deep learning approach processes the entire radiograph, potentially reducing operator-dependent variability. However, the performance is similar to the recently published work by Jones et al., which also used an end-to-end approach but on a larger, multi-institutional dataset."

## Part 5E: Future Directions: Further development, validation, or implementation steps.

### Orthopedic Examples:

- "Future work will focus on externally validating this AI model for fracture detection in a prospective, multicenter trial and integrating it into the emergency department PACS for a clinical impact study. We also plan to extend the model to identify specific fracture types and measure displacement."
- "The next steps for our prognostic model include incorporating additional biomarkers, conducting a prospective study to assess its impact on surgeon decision-making and patient outcomes, and developing a user-friendly interface for clinical use."
