# Supplementary material for: Standardised Transparent Orthopaedic Reporting and Modelling for AI (STORM‐AI)—Guidelines for reporting artificial intelligence studies in orthopaedics from the ESSKA AI Working Group
Source: J Exp Orthop. 2026 Mar 30;13(2):e70702. doi: 10.1002/jeo2.70702 (PMC13052261; doi:10.1002/jeo2.70702)
Supplement: Supplementary file 2 — ESSKA STORM‐AI Checklist. [file JEO2-13-e70702-s002.docx]

ESSKA STORM-AI Checklist

| Part | Line |
| --- | --- |
| I. TITLE AND ABSTRACT |  |
| Part 1A: Identification as a study of an AI model in orthopedics |  |
| Part 1B: Specific orthopedic problem/application addressed |  |
| Part 1C: Study objectives (e.g., developing, validating, or comparing an AI model) |  |
| Part 1D: Mention of model type (e.g., diagnostic, prognostic, segmentation) |  |
| II. INTRODUCTION |  |
| Part 2A: Scientific and clinical background, including intended use |  |
| Part 2B: Specific objectives and hypotheses, clearly stating if it's development, validation, or both |  |
| Part 2C: Intended population and setting |  |
| III. METHODS |  |
| Part 3A.i: Study Design -Prospective or retrospective |  |
| Part 3A.ii: Description of the study population and period |  |
| Part 3A.iii: Setting (e.g., multicenter, single academic hospital, public registry) |  |
| Part 3B.i: Eligibility criteria (inclusion/exclusion) for patients/data |  |
| Part 3B.ii: Source of data (e.g., PACS, EHR, orthopedic registries, wearable sensors) |  |
| Part 3B.iii: Methods of data collection and any pre-processing specific to orthopedic data |  |
| Part 3C.i: Clear definition of the outcome(s) being predicted |  |
| Part 3C.ii: How and when outcomes were assessed, including follow-up duration |  |
| Part 3C.iii: Any cut-offs used for continuous outcomes |  |
| Part 3D.i: Chosen reference standard/"ground truth" |  |
| Part 3D.ii: Rationale for choosing the reference standard |  |
| Part 3D.iii: Blinding of assessors to AI results and other clinical information |  |
| Part 3E.i: Detailed description of all input data used by the AI |  |
| Part 3E.ii: How and when input data were measured/extracted. |  |
| Part 3E.iii: Handling of missing data. |  |
| Part 3E.iv: Specific radiographic measurements used, details of segmentation, specific PROMs |  |
| Part 3F.i: Description of the AI model architecture (e.g., CNN type, layers, decision tree, etc.). |  |
| Part 3F.ii: Data pre-processing steps not covered in 3B (e.g., image augmentation techniques) |  |
| Part 3F.iii: Data partitioning: clear description of training, tuning/validation, and test sets |  |
| Part 3F.iv: Details of the training process |  |
| Part 3F.v: Approach to model selection or feature selection. |  |
| Part 3F.vi: For clinical trials: Description of the AI intervention, Integration, and protocols. |  |
| Part 3G.i: Performance metrics used. Justification for chosen metrics. |  |
| Part 3G.ii: Confidence intervals for all metrics. |  |
| Part 3G.iii: Details of internal and external validation |  |
| Part 3G.iv: Error analysis: Investigation of false positives/negatives |  |
| Part 3G.v: Comparison with existing methods or clinician performance (if applicable). |  |
| Part 3H: Explainability/Interpretability |  |
| Part 3I.i: Methods for calculating sample size (if applicable). |  |
| Part 3I.ii: Methods for handling continuous variables. |  |
| Part 3I.iii: Methods for handling missing data in model development and validation. |  |
| IV. RESULTS |  |
| Part 4A: Participant/Data Flow (Diagram showing flow of participants/data units through the study). |  |
| Part 4B: Baseline Characteristics: Demographics, clinical characteristics, and orthopedic specifics of the development and validation cohorts. |  |
| Part 4C.i: Full performance metrics on all datasets as described in 3.G. |  |
| Part 4C.ii: Calibration plots for prediction models. |  |
| Part 4E: Comparative Performance: Results of AI vs. human experts or other methods. |  |
| Part 4F: Visual examples of AI output (e.g., correctly/incorrectly classified images, segmentation overlays, heatmaps on orthopedic images). |  |
| V. DISCUSSION |  |
| Part 5A: Statement of Principal Findings: In the context of orthopedic care. |  |
| Part 5B: Clinical & Research Implications: How the findings could impact orthopedic practice, patient care, or future research. |  |
| Part 5C: Limitations: Including biases, generalizability to different orthopedic populations, settings, equipment, or implant types. |  |
| Part 5D: Comparison with Existing Literature: Strengths and weaknesses relative to other AI models or conventional approaches in orthopedics. |  |
| Part 5E: Future Directions: Further development, validation, or implementation steps. |  |

Please mention the usage of the STORM-AI guidelines in your methods section and cite our corresponding manuscript.

Comments on why certain aspects were omitted:

____________________________________________________________________________________

____________________________________________________________________________________

____________________________________________________________________________________

____________________________________________________________________________________

____________________________________________________________________________________
